# Estimation of Mediastinal Toxicities after Radiotherapy for Hodgkin Lymphoma—A Normal Tissue Complication Analysis of the HD16/17 Trial by the German Hodgkin Study Group

**DOI:** 10.3390/cancers16061168

**Published:** 2024-03-16

**Authors:** Michael Oertel, Priska Hölscher, Dominik Hering, Christopher Kittel, Michael Fuchs, Uwe Haverkamp, Peter Borchmann, Hans Theodor Eich

**Affiliations:** 1 Department of Radiation Oncology, University Hospital Muenster, 48149 Muenster, Germany; p_hoel04@uni-muenster.de (P.H.); dominik.hering@ukmuenster.de (D.H.); christopher.kittel@ukmuenster.de (C.K.); uwe.haverkamp@ukmuenster.de (U.H.); hans.eich@ukmuenster.de (H.T.E.); 2 Department of Internal Medicine, Center for Integrated Oncology Aachen Bonn Cologne, Dusseldorf, University Hospital of Cologne, 50937 Cologne, Germany; michael.fuchs@uk-koeln.de (M.F.); peter.borchmann@uk-koeln.de (P.B.)

**Keywords:** NTCP, side effects, Lyman–Kutcher–Burman, radiation therapy, Hodgkin lymphoma

## Abstract

**Simple Summary:**

Patients with Hodgkin lymphoma show excellent survival rates, emphasizing the importance of the long-term side effects of the treatment itself. This analysis aims at a cardiopulmonary risk evaluation in the context of radiation treatment for early-stage Hodgkin lymphoma using normal tissue complication probability calculations. Overall, a low cardiopulmonary risk was estimated, not exceeding 2%. Concerning radiation dose, 30 Gy is associated with consistently higher risks as compared to 20 Gy. Further individualization of treatment will be attempted in the future.

**Abstract:**

Purpose: Hodgkin lymphoma is a hematologic malignancy with excellent outcomes even in advanced stages. Consequently, the importance of treatment-associated toxicity increases. However, the exact estimation of individualized rates is difficult due to different disease extents, treatment strategies and techniques. The following analysis aims at a pre-treatment estimation of relevant mediastinal toxicities. Methods: Normal tissue complication probability calculations were used to evaluate the toxicity rates for the heart, lungs and female breast of patients undergoing radiotherapy for early-stage Hodgkin lymphoma. Overall, 45 Patients of the HD16 and HD17 trials by the German Hodgkin study group were included and risks were calculated using the Lyman–Kutcher–Burman model. Results: The median values for pericarditis, pneumonitis and fibrosis of the left or right breast were 0.0%, 0.0%, 0.7% and 0.6% in the HD16 cohort, and 0.0%, 0.1%, 1.1% and 1.0% in the HD17 cohort, respectively. Correspondingly, none of the included patients displayed any of the evaluated toxicities during clinical follow-up. The use of higher doses (30 Gy) in the HD17 cohort led to an increase in toxicity compared to the HD16 cohort (20 Gy). No significant influence of the planning target volume size or the radiation technique could be found in this study. Conclusion: Both the clinically observed and calculated toxicity rates corroborate the overall low-risk profile of radiotherapy for Hodgkin lymphoma. Further treatment individualization will be attempted in the future.

## 1. Introduction

Hodgkin lymphoma (HL) is a distinctive malignant B-cell lymphoma occurring predominantly in young adults [1]. Combined modality treatment, consisting of (immuno-) chemotherapy and radiation therapy (RT), results in an excellent long-term prognosis with clinical trials showing 5-year survival rates of >90% even in advanced stages [2,3,4]. Due to the high cure rates, therapeutic de-escalation, whilst simultaneously maintaining treatment results, has been a major focus of research interest in the last decades. This is of pivotal importance, as, for long-term survivors of HL, the treatment itself evolves as the main cause of death. In the second decade after treatment long-term toxicities, such as secondary malignancies and cardiovascular diseases, constitute the main mortality risk [5,6].

Currently, the first-line therapy consists of multiagent chemotherapy regimens like ABVD (doxorubicin, bleomycin, vinblastine, dacarbazine) or BEACOPP (bleomycin, etoposide, doxorubicin, cyclophosphamide, vincristine, procarbazine and prednisone) followed by consolidative RT [7]. Focusing on radiotherapy, there has been a constant decrease in radiation doses and field size in order to minimize dose exposure to healthy organs at risk [8,9]. The German Hodgkin Study Group (GHSG) has conducted large, randomized therapy studies on HL for several decades, the latest being the HD16 and 17 trials in patients with early-stage favorable or -unfavorable HL, respectively. Regarding RT, divergent results were obtained: whereas HD16 confirmed the necessity for consolidative RT in early-stage favorable HL after two cycles of ABVD, HD17 pointed towards positron emission tomography (PET)-guided RT in early-stage unfavorable HL and limited RT to patients with PET-positive results [2,3]. Therefore, a differentiated, individualized and risk-adapted indication for RT is advocated.

The present study aims to provide a risk evaluation of the (long-term) toxicities in mediastinal organs caused by RT. A normal tissue complication probability (NTCP) model is utilized to estimate the likelihood of radiation-induced toxicities, which allows for the comparison of estimated risk and clinical observation. Other studies have already successfully used this NTCP model [10], but some risk estimations for HL remain elusive. The extrapolated risk values are discussed in light of recent treatment trials, thereby providing new insights into personalized planning for HL.

## 2. Materials and Methods

### 2.1. Trial Design of GHSG HD16 and HD17

#### 2.1.1. HD16

The German Hodgkin Study Group (GHSG) HD16 Trial [2] (NCT00736320; EudraCT code: 2007-004474-24) was an international, randomized, phase 3 trial addressing patients with newly diagnosed Hodgkin Lymphoma (HL). Overall, 1150 patients were enrolled. Eligible criteria were a patient age between 18 and 75 years and an Ann Arbor stage I or II without any GHSG risk factors (mediastinal bulk, extranodal involvement, elevated erythrocyte sedimentation, three or more involved nodal areas). Treatment consisted of two cycles of ABVD chemotherapy (doxorubicin, bleomycin, vinblastine, dacarbazine) with subsequent randomization. In the standard arm, all patients underwent 20 Gy involved field RT (IFRT), whereas, in the experimental arm, only PET-positive patients were irradiated.

The results showed a decreased 5-year progression-free survival (PFS) with the omission of RT (86.1% without vs. 93.4% with RT). The median follow-up was 45 months.

#### 2.1.2. HD17

The randomized, phase 3 GHSG HD17 trial [3] (NCT01356680; EudraCT code: 2007-005920-34) enrolled 1100 patients in early-stage unfavorable HL between the age of 18 and 60. Following the GHSG stage definition, patients could reveal an Ann Arbor stage I-IIA with any combination of risk factors mentioned above or an Ann Arbor stage IIB with an elevated erythrocyte sedimentation rate and/or three or more nodal areas. All patients received two cycles of escalated BEACOPP (bleomycin, etoposide, doxorubicin, cyclophosphamide, vincristine, procarbazine and prednisone) and two cycles of ABVD followed by a 1:1 randomization between a standard and an experimental arm. Consolidative RT with 30 Gy as IFRT was applied to all patients in the standard arm. In the experimental arm, RT was limited to patients with PET-positive findings who then underwent an involved node RT (INRT) with 30 Gy. Comparisons between the two arms showed no significant difference regarding 5-year PFS (95.1% in the experimental arm vs. 97.3% in the standard arm). The median follow-up was 46.2 months.

### 2.2. Patient Data and RT Analysis

RT in the GHSG trials is subject to a multistep structured quality control which has been described previously [11]. In summary, treating RT facilities are invited to send treatment plans to the reference radiation oncology for central analysis. RT plans are then reviewed by an expert panel based on the initial imaging, treatment recommendation and treated RT plan and are evaluated as “according to protocol”, “minor deviation” or “major deviation”, respectively. For the present analysis, 45 patients with mediastinal radiation either above or below the tracheal bifurcation were selected from the quality analysis of HD16/17. Eligibility criteria were a digital RT planning with an available full thorax-CT scan for further contouring. Of these, 20 patients belonged to the HD16 cohort and 25 patients to the HD17 cohort. Concerning HD17, IFRT (5 patients), as well as INRT (20 patients), treatment series were included.

### 2.3. NTCP Calculations

For dosimetric analysis, the following organs at risk (OARs) were contoured centrally: lungs, heart and left and right female breast. The contouring of the OARs was reviewed and standardized by the two main authors of this work, one of whom is a senior physician in radiation oncology.

Subsequently, dose–volume histograms were generated and transformed to an equivalent uniform dose in 2-Gy fractions.

We estimated each patient’s normal tissue complication probability (*NTCP*) using the *NTCP* model proposed by Lyman–Kutcher–Burman [12], which is based on the following function:

(1) $$NTCP_{LKB}\left(D\right)=\frac{1}{2\pi }\overset{t}{\underset{-\infty }{\int }}\exp\left(\frac{-u^{2}}{2}\right)du$$

where

(2) $$t=\frac{D_{eff}-D_{50}}{m\;\cdot \;D_{50}}$$

and

(3) $$D_{eff}=\overset{M}{\underset{i=1}{\sum }}{\left(\frac{v_{i}}{V_{ref}}EQD_{2,\;i}^{1/n}\right)}^{n}$$

*u* = variable of integration, *D*_50_ = dose giving a 50% response probability, *m* = slope of the response curve, *n* = volume dependence, *M* = total number of voxels, *v*_i_/*V*_ref_ = relative volume of voxel *i* compared to the reference volume, *EQD*_2_ = equivalent dose in voxel *i* given in 2-Gy fractions.

The function is characterized by the parameter *n* for volume dependence; *m* for the steepness of the complication probability vs. dose curve; and *TD*_50_ for the dose inducing a 50% complication.

Values for the respective parameters were defined based on literature research. Regarding pericarditis and pneumonitis (grade 2 or higher), the employed values were *n* = 0.35, *m* = 0.1 and *TD*_50_ = 48, and *n* = 0.87, m = 0.18 and *TD*_50_ = 24.5, as postulated by Burman et al. [13]. For breast fibrosis, the following modified values based on the work by Mukesh et al. [14] were utilized: *n* = 0.012, *m* = 0.35 and *TD*_50_ = 132.0.

### 2.4. Statistics and Analysis

The NTCPs were calculated as described above and were analyzed with SPSS Statistics version 29.0 (IBM, Armonk, NY, USA). The OARs were analyzed separately and comparisons between the HD16 and HD17 cohort were made for each OAR. Subanalyses were conducted to evaluate the influence of both the PTV size and the RT technique utilized. To exclude confounding by PTV size, we adjusted the NTCP values by dividing by PTV and compared the relations between different OARs to the original values. To evaluate the influence of the RT technique, the NTCP values were divided into two subcohorts separating the conventional techniques (anterior-posterior-posterior-anterior (APPA), 3D-conformal radiation therapy (3DCRT)) from the intensity-modulated techniques (intensity-modulated radiation therapy (IMRT), volumetric modulated arc therapy (VMAT), tomotherapy), respectively. Comparisons between HD16 and HD17 and the radiation techniques used were executed via a Mann–Whitney U-Test with an exact two-tailed *p*-value. For all analyses, a *p*-value < 0.05 was considered statistically significant. The overall results were compared to real-world toxicity data from the GHSG study registry focusing on grade 3 and 4 toxicities as grade 1 or 2 toxicities are not documented in the study registry.

## 3. Results

### 3.1. Patient Collective

Overall, 45 patients were analyzed, the details of whom are presented in Table 1. In this patient collective, 23 were female (11 in HD16 and 12 in HD17). The median age was 35 and 26.5 for HD16 and HD17, respectively. The distributions between the Ann Arbor stages in the HD16 cohort were 3, 1, 14 and 2 for the stages IA, IB, IIA and IIB, respectively. The distributions in the HD17 cohort were 2, 1, 17 and 5 for the aforementioned stages. In accordance with the study design, none of the GHSG risk factors were present in the HD16 cohort. In the HD17 cohort, risk factors were present in 15, 14, 8 and 1 patient(s) for three or more lymph node areas, an elevated erythrocyte sedimentation rate, bulky disease and extranodal involvement, respectively.

Concerning the current analysis, the majority of patients received treatment according to protocol, but three and five patients had minor or major deviations from protocol, respectively, as evaluated by the reference radiation oncology panel. All major deviations were constituted by the insufficient coverage of an involved mediastinal region, whereas the minor deviations were diverse (one insufficient dose of 29.8 Gy, one insufficient coverage of an adjuvant region, one incorrect PTV definition).

Regarding acute toxicities, three patients showed grade 3 toxicities, including nausea, dysphagia and mucositis, following radiation treatment. No grade 4 toxicities were observed and there was no evidence of any real-world occurrences of the chronic toxicities analyzed in the NTCP model (pericarditis, pneumonitis or breast fibrosis) in the GHSG study registry.

The dose exposures of the OARs are shown in Table 2: the median RT dose exposure to the heart, lungs, left breast and right breast was 6.4, 5.4, 18.4 and 16.2 Gy in the HD16 cohort, and 20.6, 11.0, 26.2 and 24.6 Gy in the HD17 cohort.

### 3.2. NTCP Calculations

**Figure 1:**
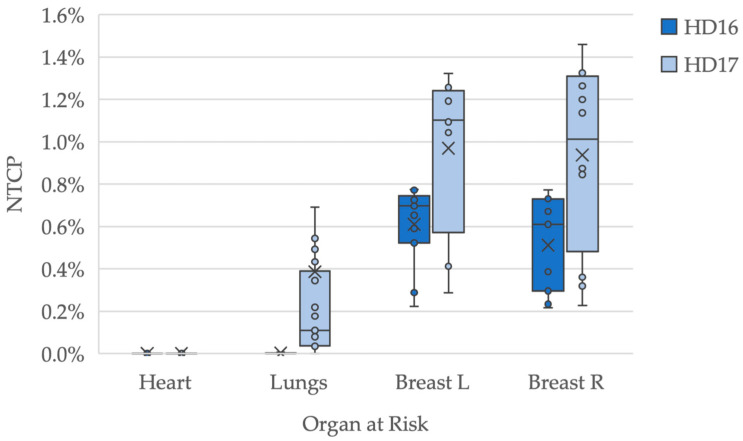
Estimated normal tissue complication probability (NTCP) for the organs at risk for HD16 and HD17 patients regarding the different (long-term) toxicities. Examined complications are pericarditis, pneumonitis and breast fibrosis, the latter being considered for female patients only. Mean values are shown with an “X”-marking. One data point at 5.2% for risk of pneumonitis in the HD17 cohort was excluded in this diagram in favor of readability.

The estimated NTCP values for the heart were 0.0% in both study groups (Figure 1, Table 3). Concerning pneumonitis, median risks of 0.0% in HD16 and 0.1% in HD17 were estimated (*p* < 0.01). Median values for HD16 and HD17 were 0.7% and 1.1% for the left breast (*p* = 0.02), and 0.6% and 1.0% for the right breast (*p* = 0.01), respectively.

**Figure 2:**
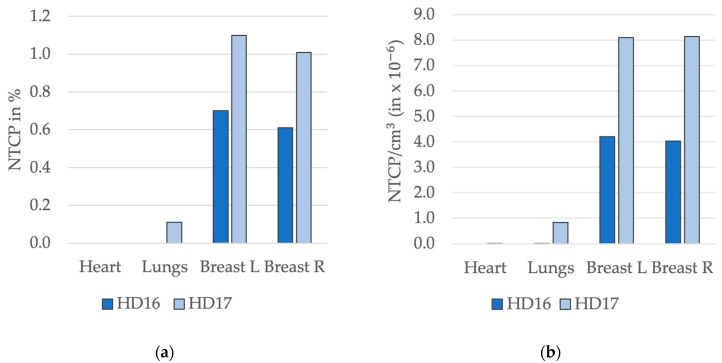
Comparison of normal tissue complication probability (NTCP) values with NTCP divided by planning target volume (PTV). (**a**) Median values of NTCP for pericarditis, pneumonitis and breast fibrosis as percentages. (**b**) Medians of NTCP in relation to PTV.

The NTCP values were adapted to the PTV size, with consistent values for the OARs between both study groups (Figure 2; Table 4). Comparing the primary NTCP medians of the cohorts, the risk of patients in the HD16 cohort developing breast fibrosis on either side was 0.6 times that of the HD17 cohort. For the heart and lungs, no relation could be estimated since the NTCP in the HD16 cohort was 0% each.

By dividing the NTCP values by the PTV size, the HD16 median for breast fibrosis on either side amounted to 0.5 times the median of HD17.

**Figure 3:**
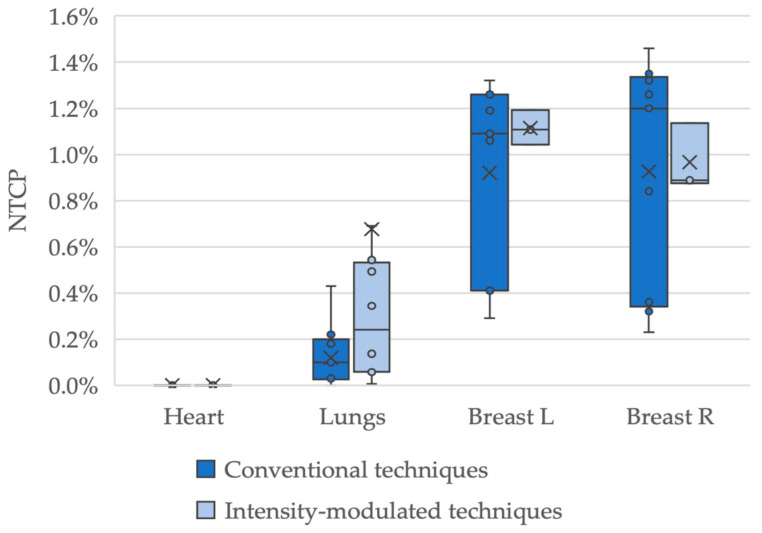
Box-plot diagram of normal tissue complication probability according to the radiation technique used. Analysis of pericarditis, pneumonitis and breast fibrosis for patients treated with conventional techniques (anterior-posterior-posterior-anterior and 3D-conformal radiation therapy) vs. intensity-modulated techniques (intensity-modulated radiation therapy (IMRT), volumetric modulated arc therapy and tomotherapy). Mean values are indicated with “X”. One outlier at 5.2% for pneumonitis in the IMRT cohort is not displayed in the figure to increase readability.

Additionally, comparisons between conventional radiation techniques and intensity-modulated treatments were performed. Only the HD17 cohort was included in this analysis as, for HD16, all but one patient was treated with a conventional APPA/3DCRT setup. In total, data on RT techniques were available for 25 (12 female) patients, 13 (9) of whom had APPA/3DCRT and 12 (3) who had intensity-modulated techniques. Of the latter group, 4 (1) were treated with IMRT, 7 (2) with VMAT and 1 (0) with tomotherapy. Figure 3 displays the comparison of the conventional techniques with the intensity-modulated techniques. The median NTCP values for the heart, lungs, left breast and right breast were 0.0%, 0.1%, 1.1% and 1.2% for the conventional techniques, and 0.0%, 0.2%, 1.1% and 0.9% for the intensity-modulated techniques. Comparing both groups showed no significant differences with *p*-values of 1.0; 0.1; 1.0; 0.9 for heart; lungs; left breast; right breast, respectively. 

## 4. Discussion

This study aims to evaluate the risk of long-term toxicities after radiation treatments in patients with HL and reveals several main findings. Firstly, it demonstrates the feasibility of estimating risks for mediastinal organs at risks in Hodgkin lymphoma via an NTCP model. Secondly, it shows a low-risk profile for all patients with median values of toxicities below 2%. This is particularly true for the heart, which was estimated to have a risk of 0%. Thirdly, these results are corroborated by clinical data, with none of the analyzed toxicities occurring during clinical follow-up. Fourthly, the radiation dose used for treatment is a key factor, as risks for pneumonitis and breast fibrosis were higher in HD17 than in HD16.

With the high survival rates achieved by modern combined-modality treatment, cardiovascular disease toxicities and secondary malignancies are the main contributors to mortality [5,6]. Radiation to the heart can induce various types of damage, including coronary heart disease, valvular disease, conducting disorders and pericardial disease [15,16,17]. These toxicities are induced by diffuse interstitial fibrosis and the accrual of myofibroblasts, leading to the narrowing of the lumen of arteries and arterioles [18,19]. The known risk factors contributing to cardiac toxicity are typical of coronary heart disease, such as high blood pressure, dyslipidemia, diabetes, adipositas and smoking, but also include higher radiation doses [20,21]; however, the substructures of the heart may display different dose–response reactions. Concerning coronary heart disease, a linear dose–response relationship was described [22], while the risk for valvular disease was increased, in particular for radiation doses above 30 Gy [23].

Radiation field size and dose are key factors determining acute and long-term toxicity in general. With the continuous decrease in field size, the radiation doses to the OARs are reduced [8,9]. Extended field radiation has been succeeded by IFRT, which has been replaced by involved site (ISRT) and INRT [18,24,25]. The present work could show that RT only holds a small risk, with median NTCP values around or less than 1%. However, a risk difference between the HD16 and the HD17 cohorts was observed. Although most patients in the HD17 cohort received radiation with a smaller field (INRT), the HD17 cohort showed a higher complication risk than the HD16 cohort, which is most likely due to the higher radiation dose. This finding is supported by the calculations provided by Hodgson et al. [26], which demonstrated a relevant risk reduction for lung and breast cancer with the use of 20 Gy IFRT instead of 35 Gy IFRT.

Furthermore, the implementation of modern IMRT allows for more conformal radiation in comparison to conventional techniques, with the opportunity to avoid higher doses of radiation to the OARs [27,28]. The concomitant increase in low-dose irradiation with IMRT is suspected to increase the risk of secondary malignancies [29]. Due to the required follow-up duration needed to acquire data on long-term toxicity rates, there is currently no general consensus favoring one treatment technique over the other, and an individual evaluation is required [7]. A study by Pepper et al. [10] compared APPA, 5-field IMRT and 7-field IMRT and analyzed the treatment plans with NTCP in regard to pneumonitis and secondary pulmonary malignancy. The least risk was found with an APPA setup (median risk for secondary malignancies: 0.35% for APPA vs. 0.44% 5-field IMRT vs. 0.53% 7-field IMRT with *p* < 0.01), although all treatment plans displayed only a very low risk. In this study, similar small risk values were estimated, although we could not find a significant difference between IMRT and APPA/3DCRT. It can be speculated that, due to limited patient numbers, the disease localizations, risk factors and stages may be imbalanced between the two groups, a drawback inherent in retrospective studies. As HD16 and HD17 recruited patients with early-stage favorable and early-stage unfavorable disease, respectively, patient characteristics varied, from an early-stage favorable HL in Ann Arbor stage IA without any of the specific risk factors, to an Ann Arbor stage I-IIA HL with any combination of the risk factors, to an early unfavorable HL in Ann Arbor stage IIB with an elevated erythrocyte sedimentation rate and/or three or more nodal areas. This is of particular importance, as previous analyses could show the association of pre-chemotherapeutic risk factors (bulk, extranodal disease) with radiation dose to the heart and lungs [30]. Consequently, the overall picture is complicated by various factors, which are mirrored by the variations in NTCP estimation.

Because of the interindividual heterogenous risk profile for long-term toxicities, the ability to determine clinically relevant risks a priori (before RT) is a powerful tool to evaluate treatment plans and choose optimal treatment solutions. This study could show the feasibility of NTCP estimations for mediastinal RT, which is only one of many applications. Previous works applied NTCP in the context of Hodgkin lymphoma as a powerful tool for individualized risk estimation and planning (Table 5). For mediastinal RT, NTCP has been used to calculate the risk of secondary malignancies [26] and to determine the risk of cardiovascular morbidity and mortality after RT [31].

In the current work, the estimated risks of long-term toxicities are supported by clinical observations. Nonetheless, the limitations of the model used have to be considered: The NTCP model proposed by Lyman relies on DVH and CT plans [32] without the consideration of clinical factors, such as age, lifestyle, smoking, preexisting diseases or family history. However, these clinical factors have proven to modify the risk for toxicities caused by radiation [33,34,35,36]. Additionally, the type and number of chemotherapy cycles used may modify the toxicity profile; the use of BEACOPP has been shown to result in a higher rate of adverse events in comparison to ABVD [37]. The most important limitation of this study is the small number of patients included and the random selection based on the availability of digital treatment plans with a potential asymmetric distribution. In particular, selection bias may occur, e.g., the patients’ median age in the presented cohort is lower, both in comparison with the epidemiology data for Hodgkin lymphoma in Germany and the study population [2,3,38]. Since the patient’s age influences the risk of radiation-induced toxicity [36], the presented values may differ from the general population. The overall low-risk numbers derived from the NTCP calculations complicate comparisons between the two study groups or concerning radiation techniques, respectively. In addition, protocol violations concerning the radiation schedule, although infrequent, may influence the dosimetric values obtained. The majority of patients analyzed have been treated with IFRT, which has been replaced by the smaller INRT or ISRT. Additionally, since the HD16 and HD17 trials were conducted, new advanced techniques, such as deep inspiration breath hold and proton radiation, have been established. Proton radiation may reduce long-term toxicity and improve target coverage [39,40], while deep inspiration breath hold has the potential to reduce the dose exposure of the heart and lungs [40].

In the future, further individualization of the treatment planning is necessary to achieve the best possible outcome for each patient. Therefore, further risk evaluations with larger patient numbers and including advanced radiation modalities are needed.

## 5. Conclusions

The presented work underlines the feasibility of using an NTCP model to estimate mediastinal toxicities in the context of HL. It demonstrates a low toxicity profile even when using the now outdated IFRT field concept. In the era of INRT and ISRT, even lower risk values are to be expected. The variety of the values derived from the NTCP calculations underlines the necessity for a personalized risk evaluation of the individual patient. Consequently, further risk stratification depending on the disease extent and radiation strategy must be undertaken, but may only be established via large databank analyses or the use of artificial intelligence.

## Figures and Tables

**Table 1:** The demographic data of the patient collective in absolute numbers and percentages (in parentheses); IFRT = involved field radiation therapy; INRT = involved node radiation therapy; IQR = interquartile range; n = absolute number of patients; RT = radiation therapy.

	n	
	HD16	HD17
Number of patients	20	25
Sex *		
Female	11 (55)	12 (48)
Male	8 (40)	12 (48)
Median age at RT	35	26.5 **
IQR	22	17.5
Risk factors		
≥3 nodal areas	0	15 (60)
Elevated erythrocyte sedimentation rate	0	14 (56)
Mediastinal bulk	0	8 (32)
Extranodal involvement	0	1 (4)
Ann Arbor Stage		
IA	3 (15)	2 (8)
IB	1 (5)	1 (4)
IIA	14 (70)	17 (68)
IIB	2 (10)	5 (20)
Radiation field		
IFRT	20	5
INRT	0	20
Radiation dose	20 Gy	30 Gy
Deviation from protocol		
None	18	19
Minor	1	2
Major	1	4
Grade 3 toxicities		
Dysphagia	0	1
Nausea	1	0
Mucositis	1	0

* One patient in each cohort excluded due to unknown gender; ** One patient excluded due to unknown age.

**Table 2:** Dose exposure to mediastinal organs at risk as estimated by radiation planning. All values are indicated in Gy. IQR = interquartile range.

		HD16	HD17
Heart	D_mean_	7.6	19.6
	D_median_	6.4	20.6
	D_IQR_	15.9	4.7
Lungs	D_mean_	5.0	10.9
	D_median_	5.4	11.0
	D_IQR_	4.2	3.2
Breast L	D_mean_	15.3	22.4
	D_median_	18.4	26.2
	D_IQR_	5.8	14.5
Breast R	D_mean_	11.9	21.2
	D_median_	16.2	24.6
	D_IQR_	14.4	18.0

**Table 3:** Estimated normal tissue complication probability (NTCP) median values for the organs at risk. IQR = interquartile range; n = number of patients. Values for median and IQR are rounded to one decimal place.

Organ at Risk	Endpoint	Study	n	Median (%)	IQR	*p*
Heart	Pericarditis	HD16	20	0.0	0.0	-
		HD17	25	0.0	0.0	
Lungs	Pneumonitis	HD16	20	0.0	0.0	<0.01
		HD17	25	0.1	0.4	
Left Breast	Breast Fibrosis	HD16	11	0.7	0.2	0.02
		HD17	12	1.1	0.7	
Right Breast	Breast fibrosis	HD16	11	0.6	0.4	0.01
		HD17	12	1.0	0.8	

**Table 4:** Comparison of median values between estimated normal tissue complication probability risk (NTCP) and NTCP in relation to the individual planning target volume (PTV; weighted NTCP). Values are rounded to one or two decimal places, respectively.

Organ at Risk	Cohort	n	Median NTCP (%)	Median NTCP (HD16/HD17)	Median NTCP/PTV	Median NTCP/PTV (HD16/HD17)
Heart	HD16	20	0.0	-	2.12 × 10^−21^	-
	HD17	25	0.0		3.73 × 10^−12^	
Lungs	HD16	20	0.0	-	5.54 × 10^−9^	-
	HD17	25	0.1		8.29 × 10^−7^	
Breast L	HD16	11	0.7	0.6	4.21 × 10^−6^	0.5
	HD17	12	1.1		8.09 × 10^−6^	
Breast R	HD16	11	0.6	0.6	4.03 × 10^−6^	0.5
	HD17	12	1.0		8.15 × 10^−6^	

**Table 5:** Overview of studies evaluating the radiation-associated complication risk for organs at risk in Hodgkin lymphoma. APPA = anterior-posterior-posterior-anterior setup; ERR = excess relative risk; EQD = organ-equivalent dose; LKB= Lyman–Kutcher–Burman; NTCP = normal tissue complication probability; QUANTEC = quantitative analyses of normal tissue effects in the clinic; RT = radiation therapy.

Author Year	Question	Endpoints	NTCP Model/ Risk Estimation	Results	Conclusion
Hodgson et al. 2007 [26]	Estimated risk difference in secondary malignancies for RT mantle 35 Gy, 35 Gy IFRT and 20 Gy IFRT	Lung cancer breast cancer	Radio- biological modeling of carcinogenesis	Compared to mantle RT, 35 Gy IFRT reduced 20-year ERR of breast cancer and lung cancer by 63% and 21%, respectively, 20 Gy IFRT reduced ERR by 77% and 57%	IFRT is predicted to have a decreased risk compared to mantle RT, but considerable interindividual variations exist
Pepper et al. 2021 [10]	Impact of RT technique on radiation induced lung disease	Pneumonitis, pulmonary secondary malignancies	LKB QUANTEC EQD-based function	According to QUANTEC parameters, pneumonitis risk is increased by 1% for 5-field IMRT and 2.6% for 7-field IMRT, in comparison to APPA (smaller risk increase with LKB model). Secondary pulmonary malignancy risk is increased by 0.1% and 0.19% for 5-field IMRT and 7-field IMRT, in comparison to APPA	Mediastinal radiation holds a very low risk for lung toxicities with APPA showing the lowest estimated risk out of the examined radiation techniques
Cutter et al. 2021 [31]	Prediction of 30-year absolute excess risk of radiation-related cardiovascular disease in a post-hoc analysis of the RAPID trial	Cardiovascular morbidity and mortality (cardiac disease and stroke)	Estimated increase in mortality rate per unit dose	Average excess in cardiovascular mortality was predicted to be 0.56% (range 0.01–6.79%), average predicted excess in incidence was 6.24% (range 0.31–31.09%) due to RT	Low predicted risk for most patients. A minority of patients receiving high doses to cardiovascular structures might profit from advanced radiation techniques or omitting RT

## Data Availability

Data relevant to this study are presented in the paper. The public deposition of data is not possible due to restrictions put in place by the Institutional Review Board.
